# The protein kinase FvRIPK1 regulates plant morphogenesis by ABA signaling using seed genetic transformation in strawberry

**DOI:** 10.3389/fpls.2022.1026571

**Published:** 2022-10-31

**Authors:** Xuexue Chen, Xiaojiao Gu, Fan Gao, Jiaxuan Guo, Yuanyue Shen

**Affiliations:** ^1^ College of Plant Science and Technology, Beijing University of Agriculture, Beijing, China; ^2^ Bei Jing Bei Nong Enterprise Management Co., Ltd, Beijing, China

**Keywords:** strawberry, *Agrobacterium*-mediated transformation of germinating seeds, reporter gene, CHLH/ABAR, RIPK1, ABA, morphogenesis, environmental response

## Abstract

A strawberry RIPK1, a leu-rich repeat receptor-like protein kinase, is previously demonstrated to be involved in fruit ripening as a positive regulator; however, its role in vegetable growth remains unknown. Here, based on our first establishment of *Agrobacterium*-mediated transformation of germinating seeds in diploid strawberry by *FvCHLH*/*FvABAR*, a reporter gene that functioned in chlorophyll biosynthesis, we got *FvRIPK1-RNAi* mutants. Downregulation of *FvRIPK1* inhibited plant morphogenesis, showing curled leaves; also, this silencing significantly reduced *FvABAR* and *FvABI1* transcripts and promoted *FvABI4*, *FvSnRK2.2*, and *FvSnRK2.6* transcripts. Interestingly, the downregulation of the *FvCHLH/ABAR* expression could not affect *FvRIPK1* transcripts but remarkably reduced *FvABI1* transcripts and promoted *FvABI4*, *FvSnRK2.2*, and *FvSnRK2.6* transcripts in the contrast of the non-transgenic plants to the *FvCHLH*/*FvABAR*-RNAi plants, in which chlorophyll contents were not affected but had abscisic acid (ABA) response in stomata movement and drought stress. The distinct expression level of *FvABI1* and *FvABI4*, together with the similar expression level of *FvSnRK2.2* and *FvSnRK2.6* in the *FvRIPK1*- and *FvABAR/CHLH*-RNAi plants, suggested that FvRIPK1 regulated plant morphogenesis probably by ABA signaling. In addition, FvRIPK1 interacted with FvSnRK2.6 and phosphorylated each other, thus forming the FvRIPK1–FvSnRK2.6 complex. In conclusion, our results provide new insights into the molecular mechanism of FvRIPK1 in plant growth.

## Highlights

Based on our first establishment of *Agrobacterium*-mediated transformation of germinating seeds in diploid strawberry (*Fragaria vesca*), we find that the strawberry *ripk1* mutant is stunted, demonstrating that FvRIPK1 is essential to plant morphogenesis by multiple abscisic acid (ABA) signaling. FvRIPK1 interacts with FvSnRK2.6, and they phosphorylate each other. Our findings provide new insights into a molecular mechanism of FvRIPK1 in plant growth and environment response.

## Introduction

Strawberry is a member of the genus *Fragaria* (*Rosaceae*), which is not only an important fruit crop worldwide but is also regarded as a model plant for studying non-climacteric fruit ripening. In the past years, much progress has been made in identification of gene function *via* traditional stable and transient transgenic systems in strawberry; however, more or less limits of both have been reported (Guidarelli and Baraldi, 2015; Haddadi et al., 2015; Zhao et al., 2019). Thus, development of a rapid, efficient, and stable transformation system is urgent in strawberry for both research and agricultural improvement.

Although strawberry leaf tissue has been widely studied to develop efficient transformation and regeneration methods through somatic tissue or callus formation (Nehra et al., 1990; El Mansouri et al., 1996; Haymes and Davis, 1997; Balokhina et al., 2000; Jimenez-Bermudez et al., 2002; Agius et al., 2003; Oosumi et al., 2006; Qin et al., 2008; Zhang et al., 2014; Song et al., 2019), the regeneration of transgenic strawberry plants takes a long time from transformation to identification (Barcelo et al., 1998; Folta and Dhingra, 2006; Haddadi et al., 2015). For example, it takes at least 15 months from transformation to gaining transgenic fruits for gene function analysis in ripening (Hoffmann et al., 2006; Miyawaki et al., 2012). In addition, although the transient transformation technology in strawberry fruits provides a rapid tool to study gene function in ripening, remarkable limits are existent in fruit transient transgenic system (Guidarelli and Baraldi, 2015; Zhao et al., 2019).

In addition to the widely used leaf disk transformation (Horsch et al., 1985; Cappelletti et al., 2015; Song et al., 2019), other tissues are also used for gene transformation (Potrykus, 1990; Hiei et al., 1994; Ishida et al., 1996; Clough and Bent, 1998; Bent, 2006; Zhang et al., 2006; Harwood, 2014; Ishida et al., 2014; Ratanasut et al., 2017; Van Eck, 2018; Song et al., 2019). The mature and immature embryo-generated calli are used to develop transgenic plants in rice, maize, wheat, and barley (Potrykus, 1990; Hiei et al., 1994; Ishida et al., 1996; Ishida et al., 2014). Notably, a non-tissue culture-based method, floral dip transformation, is widely and efficiently used in *Arabidopsis* as a model system (Clough and Bent, 1998; Bent, 2006; Zhang et al., 2006). The *Agrobacterium*-mediated transformation of germinating seeds (AMTGS) of *Arabidopsis*, another non-tissue culture approach, is also early reported (Feldmann and Marks, 1987). In recent years, the AMTGS of kenaf (*Hibiscus cannabinus*) seeds have been developed within 3 months with 6% transformation efficiency by the efficient, fast, reliable stable, non-tissue culture transgenic method (Hanana et al., 2018). Therefore, to rapidly identify gene function in strawberry, an AMTGS of diploid strawberry “Hawaii-4” (*Fragaria vesca*) is necessary to be established.

It is known that selection of a good reporter gene contributes to early identification of transgenic lines. In the past years, the H subunit of magnesium chelatase magnesium chelatase (CHLH), an enzyme involved in chlorophyll biosynthesis, is chosen as a reporter gene, in that its silencing phenotype was clearly observed through chimeric phenotypes with yellow/white leaves (Hiriart et al., 2002; Hiriart et al., 2003; Pignatta et al., 2007; Park et al., 2016). In addition, the plant CHLH has multiple functions, such as plastid-signaling trigger (Mochizuki et al., 2001), anti-sigma factor in sugar catabolism (Osanai et al., 2009), molecular circadian clock switch (Legnaioli et al., 2009), and putative abscisic acid (ABA) receptor (ABAR; Shen et al., 2006; Wu et al., 2009; Shang et al., 2010; Du et al., 2012; Zhang et al., 2013; Jiang et al., 2014; Liang et al., 2015; Bi et al., 2019). Because *CHLH* and *ABAR* are the same gene in this article, we will call it as *ABAR* in the later section. Notably, using FaABAR as a bait in a yeast two-hybrid (Y2H) assay, FaRIPK1 was recently screened, and it is a nucleus-localized and ABA-binding leu-rich repeat receptor-like protein kinase (LRR-RLKs); furthermore, the two proteins interact in the nucleus and serve as the receptor complex to regulate strawberry fruit ripening (Hou et al., 2018). However, its role in plant vegetable growth is yet unknown.

In this study, on the basis of ABAR function in chlorophyll biosynthesis, an AMTGS in diploid strawberry was first established, by which the function of RIPK1 in vegetative growth is subsequently explored for the first time. In addition, the *RIPK1* expression was investigated in the *abar* lines, in which the chlorophyll level is not significantly downregulated; meanwhile, the *ABAR* expression was also detected in the *ripk1* lines. Moreover, at the protein level, FvRIPK1 interacted with the ABA signaling component FvSnRK2.6 and phosphorylates each other. Finally, the action mechanism of the RIPK1 and ABAR was discussed.

## Materials and methods

### Plant materials and germination

The diploid strawberry Hawaii-4 (*F. vesca*) plants are grown in a green house at 25°C–28°C. The seeds were taken from ripe fruits, and the residual receptacle was removed; then the seeds were put at room temperature to dry for 1–2 days. The dried seeds are placed into a 2-ml tube and stored at 4°C for use. Seeds were sterilized by 1% sodium hypochlorite for 8 min and washed with sterile water three times. The sterilized seeds were transferred onto an Murashige and Skoog (MS) germination medium [4.43 g/L MS (PhytoTech Labs M519), 2% sucrose (Sangon, China), 0.8% agar (Sangon, China), pH 5.8] and left at 4°C for 24 h to break dormancy. The dormancy-broken seeds were treated with three manners: light culture in a light incubator (PRX-350C, Sdfu, China), dark culture wrapped with tin foil, and light culture after 2 days of dark culture. The seed culture condition in a light incubator is as a 16-h photoperiod with a light intensity of 30,000 LX (250 μmol·m^−2^·s^−1^) at 24°C. Each treatment was inoculated with 20 seeds (three repeats) for seed germination.

### Vector construction and Agrobacterium tumefaciens transformation

Two vectors were used to optimize transformation parameters, namely, a GV3101 strain carrying a pCAMBIA 1300 vector with a green fluorescent protein (GFP) reporter gene or carrying a pBI121 vector with a β-glucuronidase (GUS) reporter gene and a kanamycin (Kana) resistance gene.

The RNAi recombinant plasmid was constructed using the Gateway system, and target genes were silenced in strawberry fruit by intron splicing RNA (ihpRNA). The 465-bp *FvABAR* and 595-bp *FvRIPK1* were amplified and cloned into the pDONR™ 221 vector (Invitrogen, Beijing, China) and then into the pK7GWIWG2 (II) RR vector using the Gateway LR Clonase II enzyme (Invitrogen 11791-020, USA) combination technology. The pK7GWIWG2 (II) RR vector contains the red fluorescence protein (DsRed) reporter gene and the kanamycin resistance gene. The recombinant plasmid was transformed into *A. tumefaciens* GV3101, and a single colony was picked and placed in a Luria broth (LB) medium containing antibiotics 20 μg/ml rifampicin (Sangon, A600812, China), 50 μg/ml gentamicin (Sangon, A100304, China), and 100 μg/ml spectinomycin (Sangon, B541016, China).

The single colony of GV3101 strains (GV3101 strain of pCAMBIA 1300 and GV3101 strain of pBI121) was put into an LB medium containing antibiotics 20 μg/ml rifampicin, 50 μg/ml gentamicin, 100 μg/ml kanamycin (Sangon, A506636, China) at 28°C and 180 rpm overnight. The bacteria were collected by centrifugation at 6,000 rpm for 5 min and suspended using MS infection [4.43 g/L MS, 2% sucrose, 200 μmol/L Acetosyringone (As, Sigma-Aldrich, D134406, USA)], pH 5.8, to prepare an *Agrobacterium* infection solution. The primers used are listed in **Supplemental Table S1**.

### Optimization of parameters related to infection efficiency

The preimbibed seeds were placed in an *Agrobacterium* infection solution and cocultured at 120 rpm, 28°C for 24 h, and then the seeds were placed in an MS germination medium and wrapped in tin foil at 25°C for 3 days. Subsequently, the seeds were further placed in an MS selection medium [4.43 g/L MS, 2% sucrose, 75 mg/L kanamycin, 200 mg/L Timentin Termetin (PhytoTech Labs T869), 0.8% agar, pH 5.8]. After 3 days, GFP/DsRed fluorescent tag and GUS reporter gene were used for early screening and statistics. After 2 weeks, the seedlings with fluorescence were transplanted into quartz sand, covered with cling film for 3 days to prevent water loss, and watered with the kanamycin-added nutrient solution to allow the seedlings to grow under appropriate selection pressure at condition with a greenhouse at 24°C, light/dark 16/8 h.

To determine factors related to infection efficiency, we performed a series of experiments, namely, optical density (OD) values (OD at 600 nm, OD_600_ = 0.5, 1, 1.5, and 2), infection time (12, 24, and 36 h), and seed germination status (initial germination without white root tip, germination with white root tip, and germination with cotyledons). The optimal infection parameters were determined by investigation of GFP using stereo fluorescence microscope (ZEISS-Axiocam 506 color) and a GUS histochemical stain kit (Solarbio, G3060). Infection efficiency was calculated from the proportion of plants with GFP fluorescent protein expression or the proportion of plants with GUS expression. During the infection process, 20 seeds were placed in each Petri dish, and three replicates were performed.

### Determination of appropriate Kanamycin concentration for screening

Natural sterile seeds are cultured on the MS selection medium containing different concentrations of kanamycin with 0, 25, 50, 75 and 100 mg/L in order to determine the most suitable antibiotic screening concentration. Twenty seeds were placed in each Petri dish, and the experiment was done with three repetitions.

### Identification of transgenic plants by PCR

In order to confirm transformation of T-DNA in diploid strawberry genome, leaves of 1-month-old transgenic strawberry plants with red light were collected, and genomic DNA was extracted by Plant Genomic DNA Extraction Kit (Aidlab Biotech, DN15, China). The leaves of wild-type plants were used as control. The fragments of anti-Kana and DsRed genes were amplified by PCR. The primers used are listed in **Supplemental Table S2**.

### Stomata are sensitive to exogenous *abscisic acid* treatment

Six leaflets were selected from each seedling, and six sampled parts from each leaflet were used to determine SPAD values using a chlorophyll meter (Konica-Minolta Holdings, Inc., SPAD-502Plus). To explore the function of FvABAR in ABA response in stomata movement rather than chlorophyll synthesis, three transgenic plants with no significant difference in chlorophyll content from the control were selected to observe whether the stomata were sensitive to exogenous ABA stimulation in the buffer in an MES-KCl buffer solution [KCl 50 mmol/L, 2-(*N*-morpholino)ethanesulfonic acid (MES) 10 mmol/L, CaC1_2_ 0.1 mmol/L, pH 5.8].

ABA promotes stomata closure test: The stomata are opened by a light suspension culture in a light incubator at 24°C for 6 h, and then some leaves are incubated in an MES-KCl buffer solution for 3 h, then transferred to an MES-KCl buffer solution containing 50 μM ABA for 3 h. ABA inhibition stomatal opening test: In a 24°C light incubator, wrap the Petri dish in a dark suspension culture for 6 h to close the stomata. After that, the foil was peeled off, a part of the leaves was incubated in an MES-KCl buffer solution for 3 h, and the other part was transferred to an MES-KCl buffer solution containing 50 μM ABA for 3 h.

Observing pores with a transparent nail polish: polishing transparent nail evenly on the back of leaf, air drying for 5–8 min, stripping the lower epidermis with nail polish by hand, placing it in the center of glass slides, and then covering with coverslips. The prepared slides were observed under an optical microscope and photographed to measure the stomatal conductance. Six fields of view were selected for each treatment in the test, and five stomata apertures were measured in each field of view. Each treatment was repeated three times.

### Drought test of FvABAR-RNAi plants

Three transgenic plants and wild-type plants selected above were subjected to a drought test. Before wither, the plants were immediately transferred to a tray for watering. The experiment was repeated three times

### Real-time PCR

Total RNA from leaves of about 40-day transgenic and wild-type plants were extracted using an Omega RNA extraction kit (Omega Bio-tek, USA) according to the manufacturer’s protocols. To generate first-strand cDNA, 400 ng of total RNA was reverse-transcribed using the Trans kit (Transgen, China) according to the manufacturer’s protocols. The first-strand cDNA was used as a template for PCR amplification for real-time PCR on a LightCycler 96 real-time PCR system (Roche Diagnostics GmbH, Mannheim City, Germany) using TransStart Top Green qPCR SuperMix (Transgen, China). The reactions of 10 µl contained 5 µl qPCR SuperMix, 0.25 µl forward specific primer (10 µM; Sangon, China), 0.25 µl reverse specific primer (10 µM; Sangon, China), 2 µl cDNA template, and 2.5µl ddH_2_O. *Actin* was used as a reference gene. Relative gene expression was analyzed by LightCycler^®^ 96 SW 1.1.The primers used for real-time PCR are shown in **Supplemental Table S3**. The experiment was repeated three times.

### Kanamycin resistance test of T0 generation seeds

Twenty seeds were obtained from a single inbred T0 generation, sprinkled on quartz sand, and irrigated with kanamycin (75 mg/L). After 10 days of culture, the sensitivity of the plants to kanamycin resistance was observed, and DNA molecules were analyzed for the resistant seedlings. The primers used are listed in **Supplemental Table S2**.

### Yeast two‐hybrid assay

The Coding Sequence (CDS) of *FvSnRK2.6* was cloned into a pGBKT7 vector, and the CDS of *FvRIPK1*, *FvRIPK1-N*, and *FvRIPK1-KD* was cloned into a pGADT7 vector. The plasmids were cotransfected into the yeast strain Gold. The transfected yeast cells were placed on an SD/‐Leu‐Trp medium and incubated at 30°C for 3 days. The grown yeast was transferred to an SD/‐Leu‐Trp (‐LW) medium and an SD/‐Ade‐His‐Leu‐Trp (LWHA) medium and incubated for another 3 days.

### Firefly luciferase complementation imaging assay

The CDS of *FvSnRK2.6* was cloned into pCAMBIA1300‐NLUC and that of *FvRIPK1*, *FvRIPK1-N*, and *FvRIPK1-KD* was cloned into pCAMBIA1300‐CCLUC. The constructs were transformed into *Agrobacterium* GV3101 and coinjected into *Nicotiana benthamiana* leaves. After 48–72 h, the luciferase (LUC) signal was captured by a cold charge-coupled device camera.

### Bimolecular fluorescence complementation assay

Bimolecular fluorescence complementation (BiFC) assays were performed as described previously (Waadt et al., 2008). *FvSnRK2.6* or *FvABI1* was cloned into pSPYCE, and *FvRIPK1* was cloned into pSPYNE173. The constructions were coinjected into *N. benthamiana* leaves. After 48–72 h, the GFP signal was observed under ZEISS LSM 710.

### *In vitro* phosphorylation assay

Recombinant proteins GST-FvRIPK1, GST-FvRIPK1^K843R^, MBP-FvSnRK2.6, and MBP-FvSnRK2.6^G35R^ were purified from BL21 *Escherichia coli* and subjected to an *in vitro* phosphorylation assay. About 1 μg GST-FvRIPK1 was mixed with 2 μg MBP-FvSnRK2.6^G35R^, and the mixed proteins were incubated in a kinase buffer (2 mmol/L Tris‐HCl, pH 7.5, 1 μmol/L CaCl_2_, 0.2 mmol/L dithiothreitol, 0.5 mmol/L MgCl_2_, 0.1 mmol/L MnCl_2_), with 50 μmol/L cold ATP and 1 μCi γ‐^32^P‐ATP at 30°C for 30 min. The reactions were stopped by adding 4 μl 5× SDS loading buffer, and proteins were separated by 8% SDS‐PAGE. The protein gel was stained, decolorized, wrapped tightly with plastic wrap, and then put into the phosphor screen. Finally, radioactivity was detected by a Typhoon 9410 imager.

## Results

### Optimization of infection parameters on the basis of germination seeds

**Figure 1:**
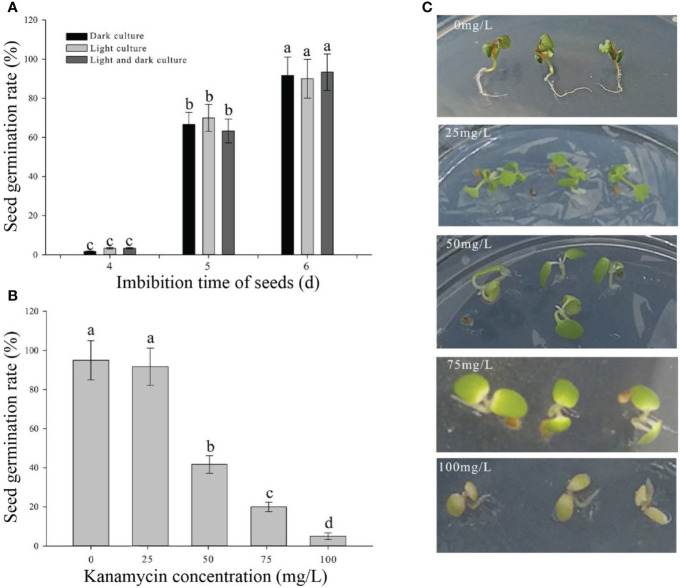
Determination of culture parameters in germination seeds and the optimal kanamycin screening concentration. **(A)** Seed germination rates in different light culture conditions and imbibing time. **(B)** Seed germination rates in different kanamycin concentrations after a 15-day cultivation. **(C)** Phenotypes in different kanamycin concentrations after a 15-day cultivation. Each treatment was treated with 20 seeds. The error bars represent the standard error (n = 3). Different letters indicate statistically significant differences at p < 0.05 as determined by Duncan’s test.

To establish rapid and stable transgenic system in strawberry, the AMTGS in diploid strawberry was first studied based on both various infection parameters and previous reports (Feldmann and Marks, 1987; Hanana et al., 2018). The seed germination rates were not significantly different under three light treatments, namely, light culture, dark culture, and light culture after dark culture for 2 days. When the seeds had imbibed for 4, 5, and 6 days, a few, half, and 90% of seeds could germinate, respectively (**Figure 1A**). Thus, light culture was used in seed germination. To confirm optimal kanamycin concentration for screening, sensitivity of the natural seeds to kanamycin is determined by adding different concentrations of kanamycin to the medium. With an increase in kanamycin concentrations at 0, 25, 50, 75, and 100 mg/L during a 15-day cultivation in an MS selection medium, the natural seeds germinated correspondingly by 95% germination seedlings with normal green seedling, 91.7% germination seedlings with light green cotyledons, 41.7% germination seedlings with no full extension cotyledons, 20% germination seedlings with yellow or white cotyledons, and 5% germination seedlings with browned or died cotyledons, respectively (**Figures 1B, C**). Thereby, 75 mg/L kanamycin in an MS selection medium was used as an antibiotic selection.

**Figure 2:**
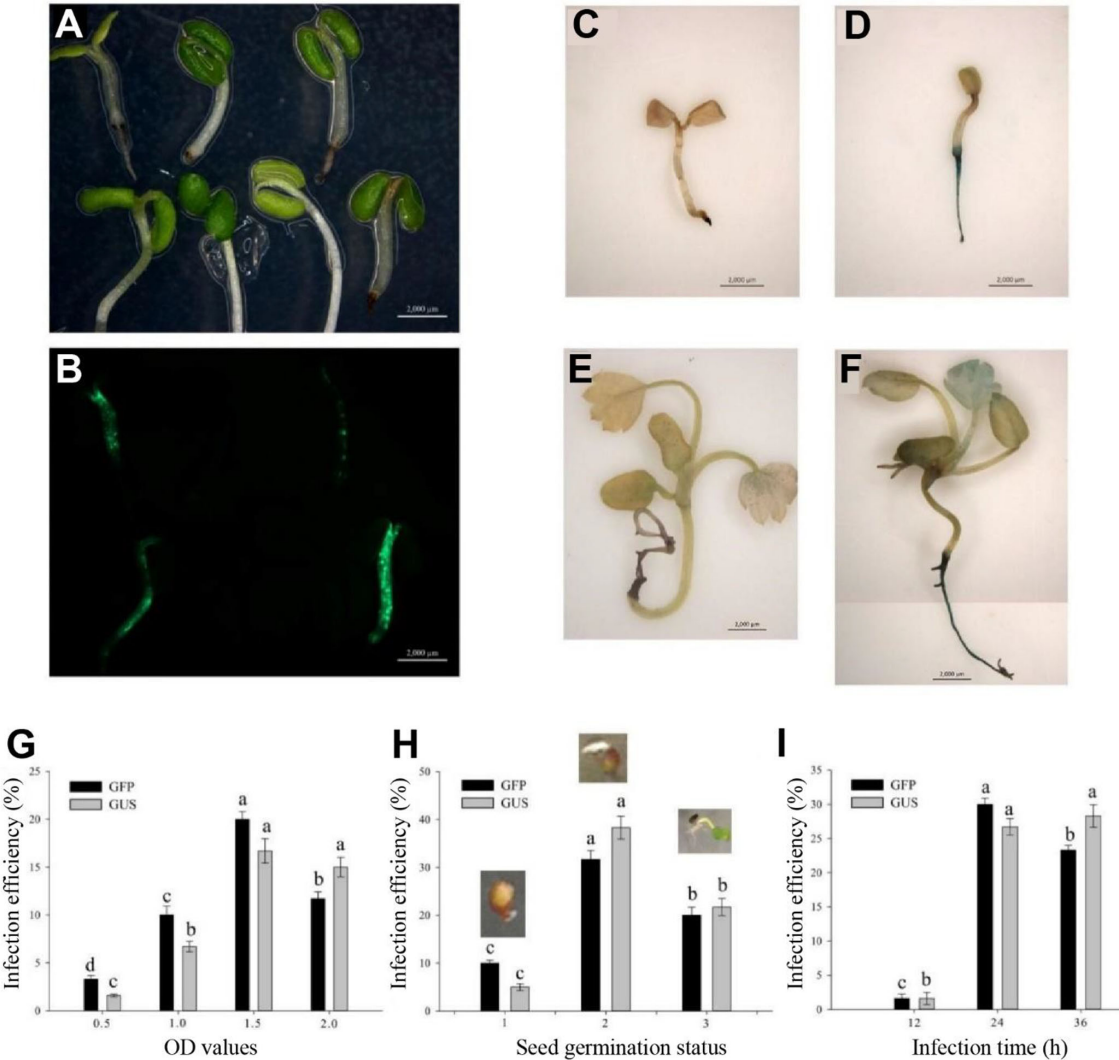
Determination of influence factors in Agrobacterium-mediated transformation of germinating seeds. **(A)** Infection seedlings after a 3-day culture. **(B)** GFP expression in the 3-day culture infection seedlings. **(C, E)** No GUS blue in the uninfection seedlings. **(D, F)** GUS blue in successfully infected seedlings. **(C, D)** Infection seedlings after a 3-day culture. **(E, F)** Infection seedlings after a 2-week culture. **(G)** Effect of optical density (OD) values on infection efficiency. **(H)** Effect of seed germination status on infection efficiency including initial germination, white root tip and green cotyledon stages. **(I)** Effect of infection time on infection efficiency. Each treatment was treated with 20 seeds. The error bars represent the standard error (n = 3). Different letters indicate statistically significant differences at p < 0.05 as determined by Duncan’s test. Bars = 0.2 cm.

After a 3-day culture in the selection medium, the GFP could be observed in the successfully transgenic plants (**Figures 2A, B**). Similarly, the not successfully infected seedlings had no GUS expression (**Figures 2C, E**), whereas the successfully infected seedlings showed blue in the roots and true leaves with GUS expression (**Figures 2D, F**). Base on the observation of both GFP and GUS expressions in transgenic seedlings, a 1.5-OD_600_ value of the *Agrobacterium* infection solution was optimum infection concentration (**Figure 2G**). The germination seeds with white root tips had the highest infection efficiency (**Figure 2H**) during the 24-h infection (**Figure 2I**).

Taken together, the optimum parameters of AMTGS in diploid strawberry are as follows: the germination seeds with white tip, 75 mg/L kanamycin in an MS selection medium, 1.5-OD_600_ value of the *Agrobacterium* infection solution, and 24-h infection in a light culture.

### Establishment of a rapid and stable transgenic system by the Agrobacterium-mediated transformation of germinating seeds by a reporter gene of FvABAR in strawberry

In addition to using the GFP and GUS as early transgenic positive selection, the *ABAR* encoding the H subunit of magnesium chelatase involved in chlorophyll biosynthesis is chosen as a reporter gene owing to its silencing phenotypes with yellow/white leaves easy to be observed (Hiriart et al., 2002; Hiriart et al., 2003; Pignatta et al., 2007; Park et al., 2016).

**Figure 3:**
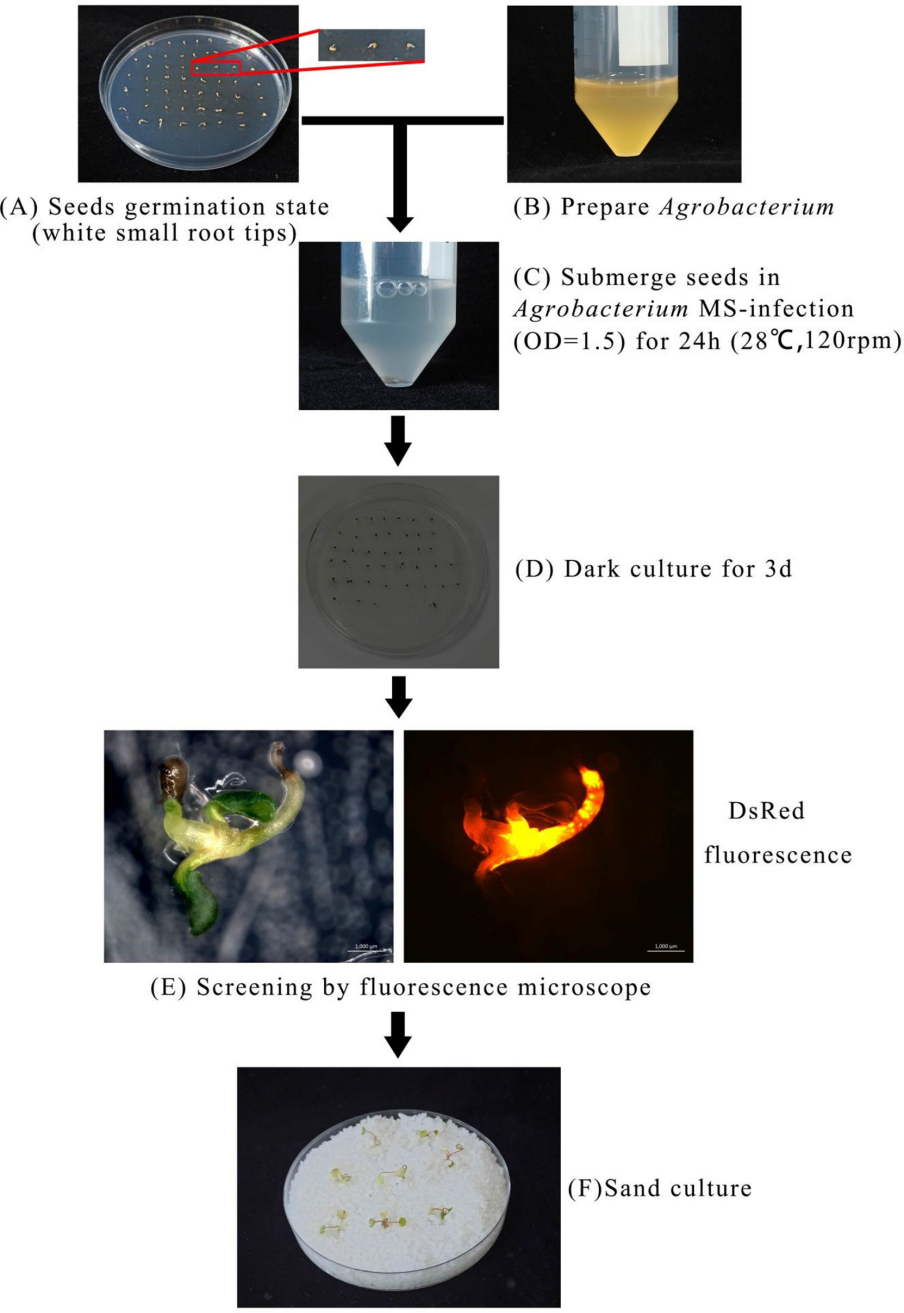
Establishment of a rapid and stable transgenic system by the Agrobacterium-mediated transformation of germinating seeds. The preimbibed seeds were placed in an Agrobacterium infection solution and cocultured at 120 rpm, 28°C for 24 h, **(A–C)** and then the seeds were placed in an MS germination medium and wrapped in tin foil at 25°C for 3 days **(D)**. Subsequently, the seeds were further placed in an MS selection medium [4.43 g/L MS, 2% sucrose, 75 mg/L kanamycin, 200 mg/L Timentin (PhytoTech Labs T869), 0.8% agar, pH 5.8]. After 3 days, GFP/DsRed fluorescent tag and GUS reporter gene were used for early screening and statistics **(E)**. After 2 weeks, the seedlings with fluorescence were transplanted into quartz sand, covered with cling film for 3 days to prevent water loss, and watered with the kanamycin-added nutrient solution to allow the seedlings to grow under appropriate selection pressure at a condition with a greenhouse at 24°C, light/dark 16/8 h **(F)**.

**Figure 4:**
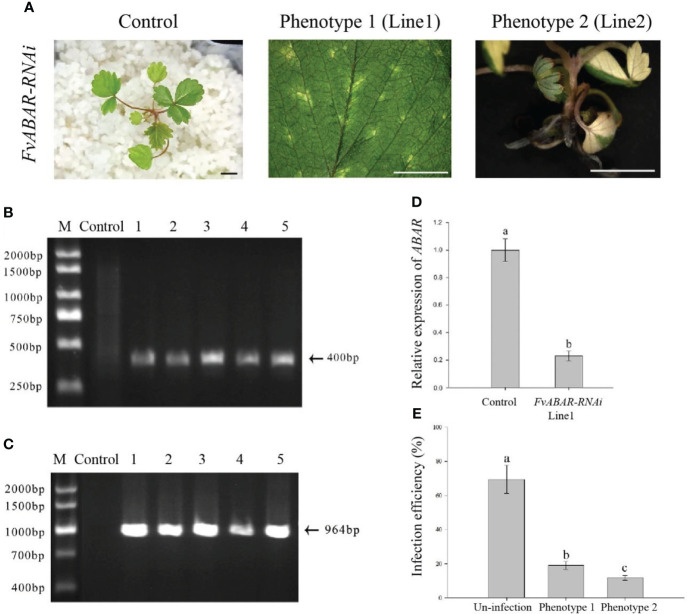
Phenotype of the FvABAR-RNAi plants in strawberry. **(A)** Loss of green color in the FvABAR-RNAi plants. **(B)** The 400-bp Kana products were detected in five FvABAR-RNAi plants (lines 1–5) but not in the wild-type plants (control) by qPCR. **(C)** The 964-bp DsRed products were detected in five FvABAR-RNAi plants (lines 1–5) but not in the wild-type plants (line 1) by PCR. The expected 400-bp Kana and 964-bp DsRed products were detected in five FvABAR-RNAi plants but not in the wild-type plants. Bars = 0.5 cm. **(D)** FvABAR gene expression of transgenic plants and wild-type plants. **(E)** Proportion of infected seedlings showing phenotype 1 and 2 plants to total infected plants.

Based on the optimal transformation conditions obtained above, we have transformed the germination seeds with white radicles of diploid strawberry “Hawaii-4” through the GV3101 strain carrying the pK7GWIWG2 (II) RR + *FvABAR* vector. After a 3-day infection under dark culture, the seeds were transferred to an MS selection medium. After 3 days, the seeds were screened by DsRed fluorescence. After 2 weeks, the fluorescent seedlings were transferred to quartz sand and watered using a nutrient solution containing 75 mg/L kanamycin. The whole processes for transformation of germination strawberry seeds were shown in **Figure 3**. One month after transplanting, in comparison to the control, the *FvABAR*-RNAi plants showed yellow spots (**Figure 4A**, line 1, phenotype 1) or half green and half white in a leaf (**Figure 4A**, line 2, phenotype 2). The PCR was performed to detect both kanamycin and *DsRed* gene fragments in the transgenic plants. The expected 400-bp Kana and 964-bp DsRed products were detected in five *FvABAR*-RNAi plants but not in the wild-type plants (**Figures 4B, C**). The RT-PCR result showed that ABAR was knocked down in FvABAR-RNAi Line1 (**Figures 4D**). And the infection efficiency of phenotype 1 and 2 was about 20% or 12% (**Figures 4E**). These results showed that on basis of the report genes including GFP, GUS, DsRed, and ABAR, a rapid and stable transgenic system by the AMTGS is success.

### Phenotypes of FvRIPK1-silencing plants by the Agrobacterium-mediated transformation of germinating strawberry seeds

**Figure 5:**
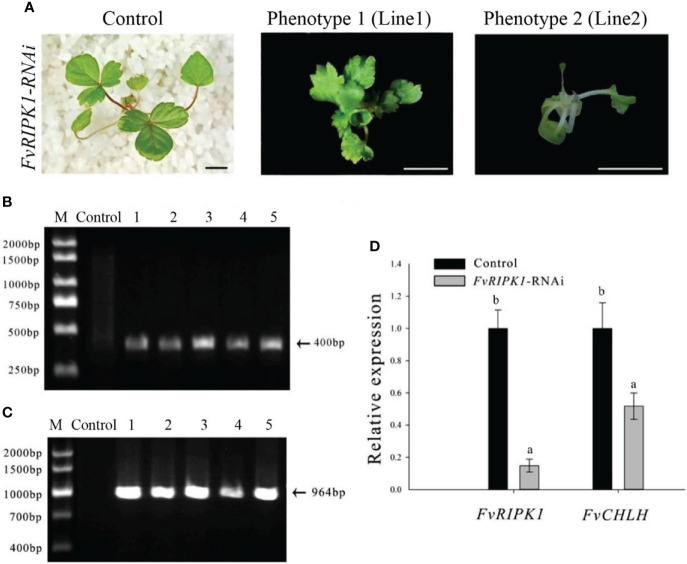
Phenotype of the FvRIPK1-RNAi plants in strawberry. **(A)** Deformed leaves with normal green color in the FvRIPK1-RNAi plants. **(B)** The 400-bp Kana products were detected in five FvRIPK1-RNAi plants (lines 2–6) but not detected in the wild-type plants (line 1) by qPCR. **(C)** The 964-bp DsRed products were detected in five FvRIPK1-RNAi plants (lines 2–6) but not detected in the wild-type plants (line 1) by qPCR. **(D)** The FvABAR expression was significantly downregulated in the FvRIPK1-RNAi plants. The error bars represent the standard error (n = 3). Different letters indicate statistically significant differences at p < 0.05 as determined by Duncan’s test. Bars = 0.5 cm.

Based on the processes of *FvABAR*-silencing transgenic plants by the *Agrobacterium*-mediated transformation of germinating strawberry seeds (**Figure 3**), the GV3101 strain carrying the pK7GWIWG2 (II) RR + *FvRIPK1* vector were used for transformation of the germination seeds. One month after transplanting, in comparison to the control, *FvRIPK1*-RNAi plants were stunted, showing curled leaves (**Figure 5A**). The phenotype of line 1 differed from that of line 2 maybe because their infected site in seeds was different. But the transgenic plants finally died and survived no more than 7 weeks after transplanting for some unknown reasons, so we cannot get the next generation to perform the assays. The PCR was performed to detect both kanamycin and *DsRed* gene fragments in the transgenic plants. The expected 400-bp Kana and 964-bp DsRed products were detected in five *FvRIPK1*-RNAi plants but not in the wild-type plants (**Figures 5B, C**). These results showed that FvRIPK1 is essential to strawberry plant growth.

Given the interaction between strawberry ABAR and RIPK1 (Hou et al., 2018), *FvABAR* expression was investigated in the *FvRIPK1*-RNAi plants. The results showed that the *FvABAR* expression levels were significantly downregulated in the *FvRIPK1*-RNAi line 1 plant, in which the *FvRIPK1* transcripts remarkably downregulated in comparison to the control (**Figure 5D**). The result indicates that *FvRIPK1* is related to *FvABAR*. Because of the *FvRIPK1-RNAi* plants with normal green leaves (**Figure 5A**) that the decrease of the *FvABAR* expression in the *FvRIPK1-RNAi* plants is mainly related to plant morphogenesis rather than chlorophyll synthesis (**Figure 5**), it may also be related to ABA signaling.

### Downregulation of *FvRIPK1* transcripts inhibits *FvABAR* expression while not vice versa, but both are involved in ABA signaling

**Figure 6:**
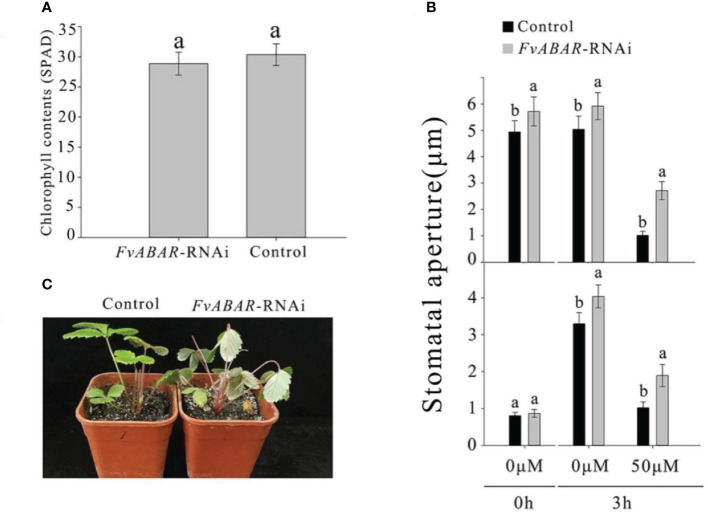
FvABAR plays a role in drought stress and stomatal movement in response to ABA. **(A)** The FvABAR-RNAi plants with no significant difference in chlorophyll contents from the wild plants. **(B)** Stomatal movement of the FvABAR-RNAi plants responsive to ABA. In the ABA-promoted stomatal closure test (top plot), the stomata are completely opened after 6 h of light culture. Then, adding 50 μM of ABA for 3 h of light culture, the stomata completely closed, and the RNAi plant showed insensitivity to ABA in stomata closure compared with the control. In the ABA inhibition stomatal opening, the stomata are completely closed after 6 h of dark culture. Then, adding 50 μM of ABA for 3 h of light culture, the stomata completely opened, and the RNAi plant showed insensitivity to ABA in stomata opening compared with the control. **(C)** Plant phenotypes of the wild-type control plants and FvABAR transgenic plants under 2 weeks of free water. The error bars represent the standard error (n = 3). Different letters indicate statistically significant differences at p < 0.05 as determined by Duncan’s test.

**Figure 7:**
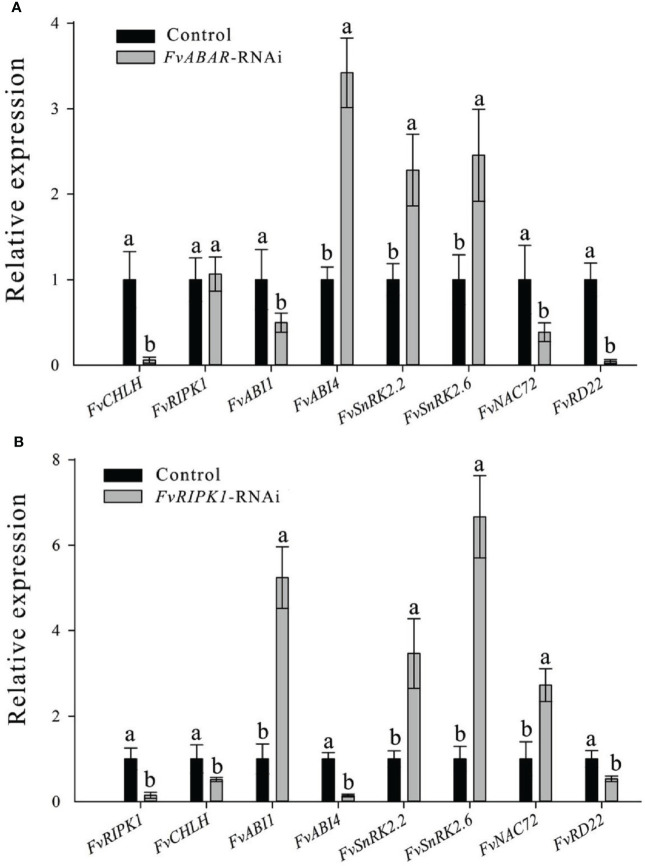
Alteration of the FvABAR or FvRIPK1 expression affects transcripts of several ABA-responsive and drought-related genes in the RNAi plants. RTPCR results showd the *FvCHLH*, *FvRIPK1*, *FvABI1*, *FvABI4*, *FvSnRK2.2*, *FvSnRK2.6*, *FvNAC72* and *FvRD22* gene expression in *FvABAR-RNAi* strawberry **(A)** and in *FvRIPK1-RNAi* strawberry **(B)**. Actin mRNA was used as an internal control. The error bars represent the standard error (n = 3). Different letters indicate statistically significantdifferences at p < 0.05 as determined by Duncan’s test.

To explore the relationship of FvRIPK1 with FvABAR at expression levels and whether both are involved in ABA signaling in strawberry, the *FvABAR*-transgenic plants had no significant decrease in chlorophyll contents (**Figure 6A**) but had ABA response in stomata movement (**Figure 6B**) and drought stress (**Figure 6C**) in comparison to the wild plants. Thus, the *FvABAR* transgenic plants were used to assess a role of FvABAR in ABA signaling including aba insensitive 1 (ABI1), aba insensitive 4 (ABI4), SNF1-related protein kinase 2.2 (SnRK2.2), SNF1-related protein kinase 2.6 (SnRK2.6), NAC domain-containing protein 72-like (NAC72), and dehydration-responsive protein RD22 (RD22) (Shen et al., 2006; Gu et al., 2017). The results showed that the downregulation of the *FvABAR* expression could not affect *FvRIPK1* transcripts but significantly promoted *FvABI4*, *FvSnRK2.2*, and *FvSnRK2.6* transcripts and significantly reduced *FvABI1*, *FvNAC72*, and *FvRD22* transcripts (**Figure 7A**). The downregulation of the *FvRIPK1* expression could reduce *FvABAR* transcripts; significantly promoted *FvABI1*, *FvSnRK2.2*, *FvSnRK2.6*, and *FvNAC72* transcripts; and significantly inhibited *FvABI1* and *FvRD22* transcripts (**Figure 7B**). The results that the downregulation of the *FvABAR* expression could not affect *FvRIPK1* transcripts, suggesting the role of FvABAR in ABA signaling was probably partially independent of FvRIPK1, whereas the downregulation of *FvRIPK1* expression reduced *FvABAR* transcripts, suggesting FvRIPK1 in ABA signaling somehow is dependent of FvABAR.

### T1 seedlings of FvABAR-RNAi plants retains kanamycin resistance

**Figure 8:**
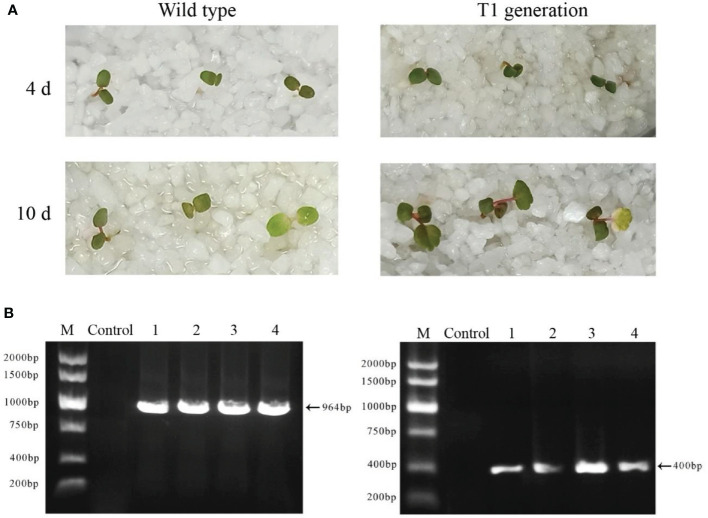
Isolation and identification of Ti-resistant plants. **(A)** The phenotype of wild-type plant seeds germinated for 4 and 10 days under the irrigation of a 75-mg/L kanamycin nutrient solution (left); the phenotype of T0 generation of FvABAR-RNAi plant seeds germinated for 4 and 10 days under the irrigation of a 75-mg/L kanamycin nutrient solution (right). **(B)** DsRed gene (left) and kanamycin resistance gene (right) of T1 generation–resistant seedlings of FvABAR-RNAi plants. Line 1 is wild-type plants, line 2 is positive control, and lines 3–5 are T1 resistant seedlings.

The seeds of wild-type plants and T1 generations of *FvABAR* transgenic plants were spread on the quartz sand soaked in the nutrient solution containing 75 mg/L kanamycin. In the presence of kanamycin, the seeds of wild-type plants cannot germinate or the cotyledons turn yellow 10 days after germination, and the true leaves could not develop. T1-resistant seedlings could germinate on quartz sand with kanamycin and continued to grow normally (**Figure 8A**). The kanamycin-resistant bands and DsRed bands were detected in T1 generations (**Figure 8B**). These results demonstrate that the transgenic plants can be obtained by seed transformation and the relative genetic characteristics may be transferred to T1 progeny.

### FvRIPK1 interacts with FvSnRK2.6 and phosphorylates each other

**Figure 9:**
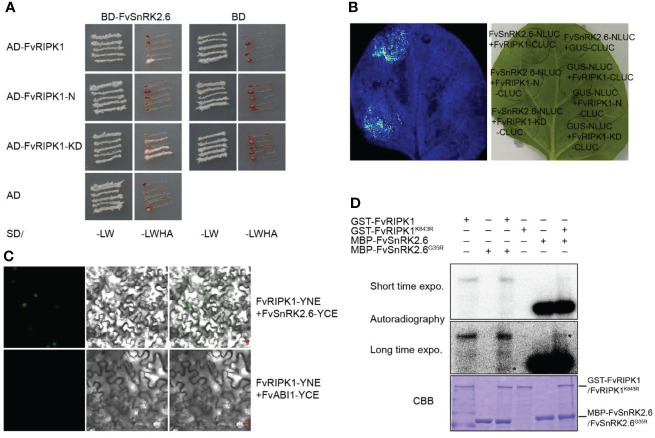
FvRIPK1 interacts with FvSnRK2.6 and phosphorylates each other. **(A)** The kinase domain (KD) domain of FvRIPK1 interacts with FvSnRK2.6 in the yeast two-hybrid (Y2H) assay. −LW, synthetic dropout medium without Leu and Trp; −LWHA, synthetic dropout medium without Leu, Trp, His, and Ade. **(B)** The luciferase complementation imaging (LCI) assay shows that FvRIPK1 or FvRIPK1-KD interacts with FvSnRK2.6. **(C)** The bimolecular fluorescence complementation (BiFC) assay indicates that FvRIPK1 interacts with FvSnRK2.6, not with FvABI1. **(D)** FvRIPK1 and FvSnRK2.6 phosphorylate each other in vitro phosphorylation assay.

As shown in the results, the downregulation of *FvRIPK1* influenced the transcription level of *FvSnRK2.6* (**Figure 7B**). However, whether FvRIPK1 has a relationship with FvSnRK2.6 at the protein level is still unknown. Based on the study before (Hou et al., 2018), we divided FvRIPK1 into two domains, namely, FvRIPK1-N-termianl (1-763 aa, FvRIPK1-N) and FvRIPK1 kinase domain (764-1154 aa, FvRIPK1-KD). First, we performed the Y2H assay to test for the interaction between FvRIPK1 and FvSnRK2.6. We found that FvRIPK1-KD was sufficient to bind FvSnRK2.6 (**Figure 9A**). Next, we used firefly luciferase complementation imaging assay for protein interaction and detected a robust luciferase signal between FvSnRK2.6 and FvRIPK1 or FvRIPK1-KD (**Figure 9B**). Moreover, FvRIPK1 interacted with FvSnRK2.6 in the nucleus, not with FvABI1 in the BiFC assay (**Figure 9C**). FvRIPK1 and FvSnRK2.6 are both protein kinases, so we next check the phosphorylation relationship between them. We got the kinase-dead form GST-FvRIPK1^K843R^ or MBP-FvSnRK2.6^G35R^ to perform the *in vitro* phosphorylation assay. The results showed that FvRIPK1 can phosphorylate FvSnRK2.6 and vice versa (**Figure 9D**), but the phosphorylation signal was weak. In all, FvRIPK1 interacts with FvSnRK2.6, and they phosphorylate each other.

## Discussion

### First establishment of rapid stable transgenic system by the Agrobacterium-mediated transformation of early germination seeds in strawberry

Strawberry is not only considered as a model plant for the *Rosaceae* family (Folta and Davis, 2006) but also regarded as an ideal model plant for studying non-climacteric fruit ripening (Li et al., 2011). Thus, in the past years, strawberry transient and stable genetic transformations have been extensively studied (Nehra et al., 1990; El Mansouri et al., 1996; Haymes and Davis, 1997; Balokhina et al., 2000; Jimenez-Bermudez et al., 2002; Agius et al., 2003; Oosumi et al., 2006; Qin et al., 2008; Zhang et al., 2014; Haddadi et al., 2015; Song et al., 2019). However, some limits exist in the present transformation system, and it is necessary to develop a rapid and stable transformation system in strawberry.

Notably, in addition to the widely used *Arabidopsis* floral dip (Clough and Bent, 1998; Bent, 2006; Zhang et al., 2006), leaf disk (Horsch et al., 1985) and the mature/immature embryos (Potrykus, 1990; Van Eck, 2018), the AMTGS of *Arabidopsis* and kenaf is ever reported (Feldmann and Marks, 1987; Hanana et al., 2018). In reference to the reports, we have also established a rapidly stable transgenic system in strawberry by the *Agrobacterium*-mediated transformation of early germination seeds using reporter genes as early (GFP and GUS) and later (DsRed and ABAR) screening as follows: (1) using seeds from ripe fruits; (2) the seeds are required to dry at room temperature; (3) the seeds are required to be fully imbibed; (4) using germination seeds with initial white root tips; (5) shaking inoculation of the initial germination seeds by the *Agrobacterium* MS infection solution (1.5 of OD_600_) for 24 h; (6) the infected seeds were darkly cultivated on the MS medium for 3 days at 24°C; (7) the dark culture seeds were transferred onto the MS selection medium to cultivate for 3 days; (8) the 3-day cultured seedlings were early screened by fluorescence; (9) after 2 weeks, the fluorescent seedlings were transferred to quartz sand.

Taken together, our first establishment of the fast (within 4 months), high-efficient (over 10% transformation efficiency), and stable transgenic system by germination seeds in strawberry is a power and useful non-tissue culture approach, which is helpful to promote the study of non-climacteric fruit ripening, strawberry genetic engineering breakthroughs, and molecular breeding development.

### The distinct and coordinate role of FvABAR and FvRIPK1 in vegetable growth and ABA signaling in strawberry

In the model plant *Arabidopsis*, ABAR serves as multiple functions, such as the H subunit of magnesium chelatase, plastid-signaling trigger, anti-sigma factor, circadian clock switch, and putative ABA receptor (Mochizuki et al., 2001; Shen et al., 2006; Legnaioli et al., 2009; Osanai et al., 2009; Wu et al., 2009; Shang et al., 2010; Du et al., 2012; Zhang et al., 2013; Jiang et al., 2014; Liang et al., 2015; Bi et al., 2019). Notably, in recent years, much progress has been made in understanding the role of ABAR in fruit ripening (Jia et al., 2010; Jia et al., 2011; Medina-Puche et al., 2014; Kadomura-Ishikawa et al., 2015; Zhang et al., 2017; Hou et al., 2018). In peach, silencing of *PaABAR* expression inhibits chlorophyll biosynthesis and fruit ripening (Jia et al., 2010; Jia et al., 2011). In strawberry, silencing of *FaABAR* expression inhibits fruit ripening (Jia et al., 2011). More recent studies have proved that FaRIPK1 (a leucine-rich repeat receptor-like protein kinase) can interact with FaABAR to regulate strawberry fruit ripening (Hou et al., 2018). However, the distinct and coordinate role of FvABAR and FvRIPK1 in vegetable growth and ABA signaling in strawberry remains unknown.

In this study, using our newly established rapid stable genetic system based on *Agrobacterium*-mediated transformation of germination seeds, the transgenic *FvABAR* lines with no significant decrease in chlorophyll contents and with ABA response in stomata movement were selected for the study (**Figure 6**), demonstrating that FvABAR plays a role in ABA signaling. We further have demonstrated a main role of FvRIPK1 in plant morphogenesis rather than chlorophyll synthesis (**Figure 5**). Interestingly, the downregulation of the *FvABAR* expression could not affect the *FvRIPK1* expression, but significantly reduced the *FvABI1* expression and promoted the *FvSnRK2.2* and *FvSnRK2.6* expressions (**Figure 7**), suggesting that FvABAR plays a role in ABA signaling independent of FvRIPK1. The downregulation of the *FvRIPK1* expression significantly inhibited the *FvABAR* expression and also significantly promoted the *FvSnRK2.2* and *FvSnRK2.6* expressions (**Figure 7**), suggesting that the role of *FvRIPK1* is somehow dependent of *FaABAR*. Notably, the *FvSnRK2.2* and *FvSnRK2.6* expressions were both significantly upregulated in the *FvABAR*- and *FvRIPK1*-RNAi plants (**Figure 7**), suggesting that FvSnRK2.2 *and* FvSnRK2.6 play an important role in coordinate-regulation of ABA signaling. Indeed, a previously report finds a link between ABAR and SnRK2.6/OST1 in guard cell signaling in response to ABA in *Arabidopsis* (Liang et al., 2015). It is also previously reported that FaSnRK2.6 is a negative regulator of strawberry fruit development (Han et al., 2015). Thus, the higher expression of *FvSnRK2.6* in the *FvABAR*- and *FvRIPK1*-RNAi promoted a negative ABA signaling. In addition, we found that FvRIPK1 interacts with FvSnRK2.6 in the nucleus, and they phosphorylate each other (**Figure 9**). But the phosphorylation sites and the regulatory mechanism of the complex FvRIPK1–FvSnRK2.6 in fruit ripening are needed to be identified. Together, the higher expression of *FaABI1* in the FvRIPK1-RNAi plants (**Figure 7**) suggests that FvABI1 plays a distinct role in FvRIPK1-mediated ABA signaling.

**Figure 10:**
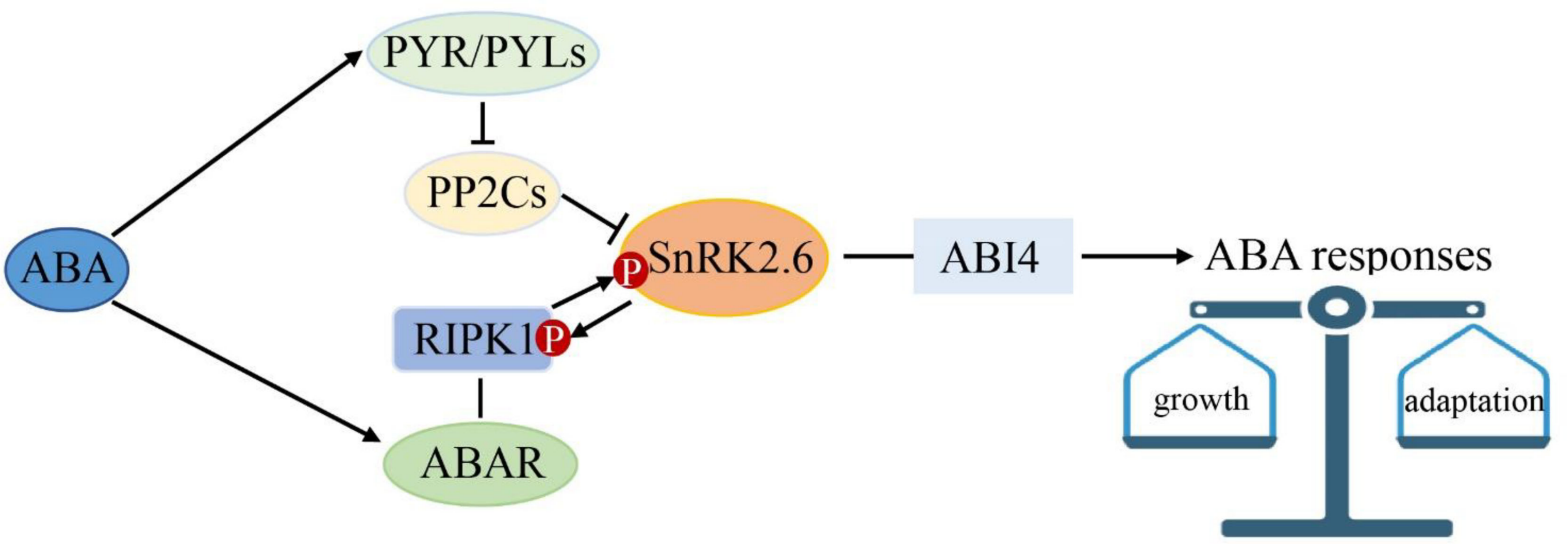
SnRK2.6 is the center crosstalk in multiple abscisic acid (ABA) signaling. ABA is perceived by PYR1 and the ABA receptor complex of ABAR/RIPK1. SnRK2.6 is the shared component between the classical “ABA-PYR1/PYLs-PP2Cs-SnRK2s-ABI4” and the “ABA-ABAR/RIPK1-SnRK2s-ABI4” ABA signaling pathways. This model provided new insights into a molecular mechanism of ABA in plant growth and environment response.

In the context, we propose a model for ABA multiple signaling linked by SnRK2.6 in strawberry (**Figure 10**). ABA is perceived by PYR/PYLs and the putative ABA receptor complex of ABAR/RIPK1. SnRK2.6 is the shared component between the classical “ABA-PYR-PP2Cs-SnRK2.6-ABI4” and the “ABA-ABAR/RIPK1-SnRK2.6/ABI4” signaling pathways. We hypothesize that the two ABA signaling pathways help plants to keep a balance between growth and adaptation to the environment. Given that RIPK1 is essential to plant morphogenesis, this model provided new insights into a molecular mechanism of ABA in plant growth and environment response.

## Data availability statement

The original contributions presented in the study are included in the article/**Supplementary Material**. Further inquiries can be directed to the corresponding authors.

## Author contributions

X-XC, X-JG and FG do experiments, Y-YS and J-XG wrote the manuscript. All authors contributed to the article and approved the submitted version.

## Funding

This study was supported by the National Natural Science Foundation of China (Projects 32072516 and 32030100) and the Beijing Natural and Science Foundation Scientific Research Key Program of the Beijing Commission of Education (Grant No. KZ202010020028).

## Conflict of interest

Author XC was employed by Bei Jing Bei Nong Enterprise Management Co., Ltd.

The remaining authors declare that the research was conducted in the absence of any commercial or financial relationships that could be construed as a potential conflict of interest.

## Publisher’s note

All claims expressed in this article are solely those of the authors and do not necessarily represent those of their affiliated organizations, or those of the publisher, the editors and the reviewers. Any product that may be evaluated in this article, or claim that may be made by its manufacturer, is not guaranteed or endorsed by the publisher.
