# Supplementary material for: The protein kinase FvRIPK1 regulates plant morphogenesis by ABA signaling using seed genetic transformation in strawberry
Source: Front Plant Sci. 2022 Oct 31;13:1026571. doi: 10.3389/fpls.2022.1026571 (PMC9659869; doi:10.3389/fpls.2022.1026571)
Supplement: Supplementary file 1 [file DataSheet_1.pdf]

Supplementary Data

Table S1. Primers used for vector construction.

Primer name	Forward primer (5'-3')	Reverse primer (5'-3')
FvCHLH-RNAi	GGGGACAAGTTTGTACAAAAAAGC	GGGGACCACTTTGTACAAGAAAGC
	AGGCTTCGCTGCCAACAATCCATCT	TGGGTCTCTTCCAACTGTCTCAGCCA
FvRIPK1-RNAi	GGGGACAAGTTTGTACAAAAAAGC	GGGGACCACTTTGTACAAGAAAGC
	AGGCTTCCGAAAGCCCTAAAACCTCC	TGGGTCCGCAACTCTCAACGAAACC

Table S2. Primers used for identification of positive plants.

Primer name	Forward primer (5'-3')	Reverse primer (5'-3')
Kana	CGATAGAAGGCGATGCGCTG	CGCTTGATCCGGCTACCTG
DsRed	cgccccttggtcaccttcagcttcac	caatgcagtgggacccacggttc

Table S3. Primers used for gene expression analysis by qRT-PCR.

Primer name	Forward primer (5'-3')	Reverse primer (5'-3')
FvCHLH	TGGGTCCCCTGATAAC	CCAAATCCCACTGTCC
FvRIPK1	CTGCACAAGTTCAAGGG	AAGGTCCAGATACTCCAAG
FvABI1	CAAGAGCCATTCTTTGTCGT	TGGAATAATCCAGGGTTTCA
FvABI4	AGACGACACTTCCCCACC	GCCACCTTTGCCCTTG
FvSnRK2.2	CCAGATAGTGTGCGAGTTTCAG	ACTTCCTCCTTCCTTCATTTCC
FvSnRK2.6	GCTACACTCGCAACCAAAATC	ACCCCACAAGACCAGACATC
FvRD22-	CAAAGGGAACCACCAT	GGCAGTAGAAGACAGCGTA
FvNAC72	CGTTTTACCTCCCACC	AAATCGTAGCCGTTGTC
FvACTIN	GCCAACCGTGAGAAGATG	TCCAGAGTCAAGAACAATACCAG
